# HIF-1α knockdown by miRNA decreases survivin expression and inhibits A549 cell growth *in vitro* and *in vivo*

**DOI:** 10.3892/ijmm.2013.1405

**Published:** 2013-06-04

**Authors:** WEI LI, YU-QING CHEN, YUAN-BING SHEN, HONG-MEI SHU, XIAO-JING WANG, CHENG-LING ZHAO, CHANG-JIE CHEN

**Affiliations:** 1 Department of Respiration, First Affiliated Hospital, Bengbu Medical College, Provincial Key Laboratory of Respiratory disease in Anhui, Bengbu, Anhui 233004, P.R. China; 2 Department of Biochemistry, First Affiliated Hospital, Bengbu Medical College, Provincial Key Laboratory of Respiratory disease in Anhui, Bengbu, Anhui 233004, P.R. China

**Keywords:** Hypoxia-inducible factor-1α, miRNA, survivin, lung cancer

## Abstract

The present study examined the downregulation of survivin expression by hypoxia-inducible factor-1α (HIF-1α) miRNA and its effect in the inhibition of A549 cell growth *in vitro* and *in vivo*. Survivin expression, apoptosis, proliferation and migration under normoxic and hypoxic conditions were assessed by standard methods. Cotransfection and chromatin immunoprecipitation were used to observe the effects of HIF-1α on survivin transcription. HIF-1α knockdown in A549 cells were injected into nude mice to examine survivin expression and suppression of tumorigenicity. Transfection of A549 cells with HIF-1α miRNA led to decreased expression of HIF-1α and survivin mRNA and protein. Survivin overexpression is mediated by HIF-1α by direct binding to a putative binding site in the *survivin* core promoter. HIF-1α-miRNA induced apoptosis and inhibited proliferation of A549 cells under hypoxic, but not normoxic, conditions, whereas transfection by survivin expression vectors partly rescued the apoptotic phenotype and revived cell proliferation under hypoxic conditions. However, cell migration was substantially suppressed by HIF-1α silencing under normoxic and hypoxic conditions. After A549 cells were xenografted in nude mice, survivin expression in mice treated with HIF-1α miRNA was downregulated, and tumor growth was significantly inhibited. Silenced HIF-1α gene expression induced apoptosis and suppressed growth of A549 cells by downregulating survivin expression *in vitro* and *in vivo*. Our results also provide a basis to target the HIF-1α pathway in lung cancer therapy.

## Introduction

Lung cancer is the most common malignancy worldwide, with approximately 1.3 million new cases and 300,000 deaths each year, as estimated by the World Health Organization (1). As non-small cell lung cancer (NSCLC) accounts for 80–85% of all lung cancer cases, understanding the pathogenic mechanism of NSCLC is critical (2).

Survivin, a member of the inhibitor of apoptosis protein (IAP) family, is a key regulator of mitosis and programmed cell death. Although minimally expressed in normal adult tissues, survivin is highly expressed in most human tumors, such as melanoma and cancer of the lung, esophagus, stomach, intestine, pancreas and breast (3). Of note, survivin is associated with tumor progression, angiogenesis, poor patient prognosis, resistance to radiation and drug treatments, and increased rate of cancer relapse (3–5). Several pharmacological and environmental stimuli, such as UVB exposure, chemotherapeutic agents, hypoxia and vascular injury, can increase survivin expression (6); survivin has also become a therapeutic target and a potentially important prognostic marker for numerous types of tumor.

Hypoxia is a unique microenvironment in solid tumors, including lung cancer. Since vasculature in tumors is dysfunctional, rapid growth of tumor cells results in insufficient oxygen supply (7). Hypoxia is associated with increased malignancy, resistance to therapy and distant metastasis (8–10). Hypoxia-inducible factor-1 (HIF-1), a master transcription factor of oxygen-regulated genes, mediates a wide range of cellular and physiological adaptive responses to changes in oxygen tension (11). HIF-1 is composed of two subunits, HIF-1α and HIF-1β (12). HIF-1α is the main active subunit, which can induce a vast array of gene products that control energy metabolism, neovascularization, survival and cell migration and is a strong promoter of tumor growth (13). In our previous study, we found that HIF-1α and survivin were widely expressed in both A549 cells and fresh NSCLC tissue samples, and increased significantly in hypoxia compared with normoxia (14). This finding is consistent with other studies that show survivin expression is induced by hypoxia (15). We speculate that HIF-1α could become an important target for lung cancer therapy. Herein, we showed HIF-1α expression knocked down by miRNA to inhibit proliferation and promote apoptosis under hypoxia, to increase cell migration in both hypoxia and normoxia, to reduce survivin expression and to trigger apoptosis *in vitro* and *in vivo*. We also confirmed that HIF-1α mediates survivin overexpression by direct binding to the *survivin* promoter region.

## Materials and methods

### Cell lines and culture conditions

Human lung adenocarcinoma cell lines were obtained from the Cell Culture Center, Chinese Academy of Medical Sciences (Shanghai, China). A549 cells were maintained in Ham’s F12 medium supplemented with 10% fetal bovine serum. Cells were incubated under normoxic (20% O_2_) or hypoxic (cobalt chloride, a hypoxia-mimicking agent, the maximum expression of HIF-1α was with 150 μmol/l CoCl_2_) conditions.

### HIF-1α miRNA construct and cotransfection with survivin expression vectors

For the miRNA construct, one target sequence (5′-GCAGGTCATAGTTTTGGCCACTG-3′) was selected corresponding to the open reading frame of the human HIF-1α gene (NM-001530). The construct containing a scrambled sequence (5′-CGTGGAGACGTTTTGGCCACTGA-3′) (Scrambled) was also included as a negative control; it has no significant homology with human gene sequences. They were synthesized by Invitrogen and inserted into pcDNA6.2-GW/EmGFP eukaryotic expression vectors to construct miRNA or negative control vectors, which were termed HIF-1α-miRNA and Scrambled, respectively. For gene transfection, 2×10^5^ cells per well were set into 6-well plates and grown overnight until they were 50–80% confluent. Plasmids HIF-1α-miRNA and Scrambled were transfected into A549 cells by Lipofectamine 2000 reagent (Invitrogen) as per the manufacturer’s instructions. Cells were subcultured at a 1:5 dilution in 300 mg/ml G418-containing medium. Positive stable transfections were selected and expanded for further study. The pCLEN plasmid encoding full-length survivin was a kind gift from Dr Feng Qian (Department of Pharmacology, University of Illinois, Chicago, IL, USA). Cells were transfected twice with 2 μg of expression vector or empty pCRII-TOPO control (Invitrogen) 6 and 24 h after HIF-1α-miRNA transfection (described above) using the FuGENE 6 Transfection Reagent (Roche Diagnostics) as per the manufacturer’s recommendations. Cells were harvested 24 h after transfection for western blotting.

### Cotransfection of survivin promoter-luciferase reporter vectors and HIF-1α expression vectors

Constructs were removed from pGL3-basic by restriction endonuclease *Mlu*I/*Hin*dIII, following procedures described in our previous study (14). Reporter vectors were constructed by T4 DNA ligase, known as pGL3-SVP-230-luc. The plasmid encoding HIF-1α, known as pcDNA3-HIF-1α, was a kind gift from Dr Feng Qian. Cells were plated at 5×10^5^ cells per well in 6-well dishes and allowed to settle overnight. The following morning, cells were cotransfected with constructs (pLuc-surP-230 and pcDNA3-HIF-1α or pcDNA3) using Lipofectamine 2000 according to the manufacturer’s protocols; 30 h after transfection, cells were harvested and lysed with 1X lysis buffer (Promega); 20 μl of cell extract was then assayed for luciferase activity using the Dual-Luciferase assay kit (Promega) according to the manufacturer’s instructions. Relative levels of reporter gene expression were expressed as ratios of firefly luciferase activity to Renilla luciferase (LU/RL).

### Chromatin immunoprecipitation (ChIP)

To demonstrate direct binding of HIF-1α protein to the *survivin* promoter region in A549 cells under both normoxic and hypoxic conditions, ChIP was performed using the ChIP-IT Express kit (Active Motif) according to the manufacturer’s protocols. Briefly, A549 cells were transfected with pcDNA3-HIF-1α or pcDNA3 prior to fixation with 1% formaldehyde for 10 min. Cells were then washed, lysed, and sonicated to reduce DNA lengths to a range of 300–600 bp. The HIF-1α/DNA complexes were incubated with mouse antibody against HIF-1α (Santa Cruz Biotechnology, Santa Cruz, CA, USA), or normal mouse IgG (Santa Cruz Biotechnology) for 18 h at 4°C. The immune complexes were precipitated, eluted, reverse-crosslinked and treated with proteinase K. The resulting DNA samples were amplified using primers for the putative HIF-1α site in the human *survivin* promoter region confirmed by our previous study (15) (F, 5′-GCGTTCTTTGAAAGCAGT-3′ and R, 5′-ATCTGGCGGTTAATGGCG-3′).

### Reverse transcription-PCR

Total RNA was isolated using TRIzol reagent (Invitrogen) according to the manufacturer’s instructions. Concentration of total RNA was detected by UV spectrophotometry. RT-PCR was performed by the two-step method. Synthesis of cDNA was performed using the cDNA Synthesis kit (Thermo, Shanghai, China). The PCR reaction conditions were: 95°C for 5 min, 94°C for 30 sec, 56°C for 30 sec, 72°C for 30 sec for 35 cycles; the total volume was 20 μl. For quantitative analysis of *HIF-1α* and *survivin* mRNA, expression of the housekeeping gene *GAPDH* was used as an internal standard. The primers used in this study were: F, 5′-AGCCAGACGATCATGCAGCTACTA-3′ and R, 5′-TGTGGTAATCCACTTTCATCCATTG-3′ for *HIF-1α* (167 bp); F, 5′-AGGTCATCTCGGCTGTTCCTG-3′ and R, 5′-TCATCCTCACTGCGGCTGTC-3′, for *survivin* (147 bp); and F, 5′-GGTCTCCTCTGACTTCAACA-3′ and R, 5′-AGCCAAATTCGTTGTCATAC-3′ for *GAPDH* (375 bp). Primers were synthesized by Shanghai Sangon Biological Engineering Technology & Services Co., Ltd. PCR fragments were separated and visualized in 20 g/l agarose gels stained with ethidium bromide. Semi-quantitative analysis was performed with Gis gel analysis software (Shanghai, China). All experiments were performed in triplicate. Ratios of photo-density of RT-PCR products of target genes and *GAPDH* were used to identify expression intensity of target genes.

### Western blot analysis

Tumor tissues were ground and sonicated with supersonic lytic buffer that contained 50 mmol/l NaH_2_PO_4_, 100 mmol/l Tris-HCl, 250 mmol/l NaCl, 100 mg/l PMSF, 1 mg/l aprotinin, pH 8.0, and then centrifuged at 12,000 × g for 40 min. A Bio-Rad standard curve was used to determine protein concentration in each lysate. Loading buffer was added to each lysate, which was then boiled for 5 min and electrophoresed by SDS-PAGE. The proteins were mixed with 2X loading buffer to the same volume prior to electrophoresis. After transferring onto nitrocellulose, proteins were incubated with antibodies (anti-HIF-1α, anti-survivin and β-actin, purchased from Santa Cruz Biotechnology), and then with peroxidase-conjugated secondary antibody (Santa Cruz Biotechnology). Detection was performed with an enhanced chemiluminescence agent. Analysis was performed with Bandscan analysis software (Sterling, VA, USA). All experiments were carried out in triplicate. Ratios of HIF-1α, survivin and β-actin proteins were used to identify expression intensity.

### Cell viability CCK-8 assay

After G418 selection for 4–5 weeks, HIF-1α-miRNA, Scrambled and untreated cells were exposed to CoCl_2_ at 150 μmol/l in 96-well plates for 24, 48 and 72 h. Cell viability was detected by Cell Counting Kit-8 (CCK-8). Following treatment, 10 μl of CCK-8 solution was added to each well; the 96-well plate was continuously incubated at 37°C for 1 h, then OD values for each well were read on a microplate reader (Multiskan, Thermo, USA) at 450 nm to determine cell viability. The assay was repeated 3 times. Cell viability was calculated as follows: % cell viability=[(OD_experiment_ - OD_blank_)/(OD_control_ - OD_blank_)] ×100%.

### FACS assay

Transfected cells and control cells in the log growth phase were harvested by trypsinization at 48 h under normoxic and hypoxic conditions for flow cytometry. Apoptotic cells in early and late stages were detected using an Annexin V-FITC Apoptosis Detection kit from BioVision (Mountain View, CA, USA). In brief, 5.0×10^5^ cells were transfected with oligos at various concentrations in the presence of Lipofectin (7 mg/ml) for 48 h. Media and cells were then collected. Cells harvested by centrifugation were washed with serum-free media and re-suspended in Annexin V Binding Buffer (500 ml); Annexin V-FITC (5 ml) and then propidium iodide (PI; 5 ml) were added. Samples were incubated in the dark for 5 min at room temperature (25.8°C) and then analyzed using a Becton Dickinson FACSCalibur (Ex=488 nm; Em=530 nm). Cells positive for Annexin V-FITC alone (early apoptosis) and for Annexin V-FITC and PI (late apoptosis) were counted. Each assay was repeated 3 times.

### Transwell invasion assay

Transwell filters (Costar, USA) were coated with Matrigel (3.9 mg/ml, 60–80 ml) on the upper surface of the polycarbonic membrane (diameter: 6.5 mm; pore size: 8 mm). After incubating at 37°C for 30 min, Matrigel became solidified and served as the extracellular matrix for tumor cell invasion analysis. Harvested cells (1×10^5^) in 100 ml of serum-free Ham’s F-12 were added into the upper compartment of the chamber. A total of 200 ml conditioned medium derived from A549 cells was used as a source of chemoattractant and placed in the bottom compartment of the chamber. After 24 h of incubation at 37°C with 5% CO_2_, the medium was removed from the upper chamber. Non-invading cells on the upper side of the chamber were scraped off with a cotton swab. Cells that had migrated from Matrigel into pores of the inserted filter were fixed with 100% methanol, stained with hematoxylin, mounted and dried at 80°C for 30 min. The number of cells invading through the Matrigel was counted in 3 randomly selected visual fields each from the central and peripheral portions of the filter, using an inverted microscope at ×200 magnification. Each assay was repeated 3 times.

### Subcutaneous tumor model

Male immune-deficient nude mice (4 weeks old) (BALB/c-nu) were purchased from Shanghai Slac Laboratory Animal Co., Ltd., bred at the facility of laboratory animals, Bengbu Medical College, and housed in micro-isolator individually ventilated cages with water and food. All experimental procedures were carried out according to the regulations and internal biosafety and bioethics guidelines of Bengbu Medical College and the Bengbu Municipal Science and Technology Commission. Mice were divided into 3 groups of 8 mice each. Each mouse was simultaneously injected subcutaneously with 1×10^7^ of A549 cells transfected with HIF-lα miRNA, Scrambled miRNA (control) or A549 cells untreated. Mice were monitored daily and all formed subcutaneous tumors. Tumor dimensions of 3 groups were measured every day with a sliding caliper using the formula: volume = length × width^2^ ×0.52. When tumor volume reached ~50 mm^3^, tumor dimensions were measured every three days. At 58 days after injection, tumors were surgically removed and weighed. Animals were monitored by general observation and determination of body weight until they were euthanized.

### TUNEL assay

Tumor tissues were fixed with 10% formalin for 4 h and then embedded in paraffin. Slices were deparaffinized in water and placed in 3% H_2_O_2_ for 10 min at room temperature. The TUNEL assay was carried out according to the manufacturer’s instructions (Beyotime Institute of Biotechnology, Beijing, China). Positive results showed brown nuclear staining.

### Statistical analyses

All assays were repeated 3 times to ensure reproducibility. For comparisons of the 3 assays and between groups ANOVA and Student’s t-test were used, respectively. All tests were performed using SPSS 11.5. Results are displayed as the means ± SD. P<0.05 was considered to indicate a statistically significant difference.

## Results

### Effect of HIF-lα-miRNA on survivin expression in A549 cells

**Figure 1:**
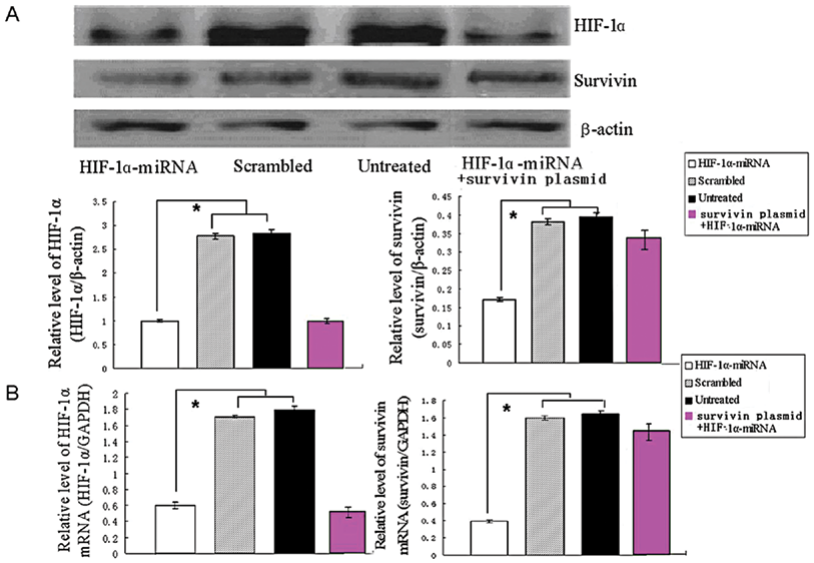
HIF-1α expression knocked down by miRNA plasmids in A549 cells under hypoxia. (A) Western blot analysis of HIF-1α and survivin protein expression using total protein extracted from cells. (B) *HIF-1α* and *survivin* gene expression were measured by RT-PCR using total RNA isolated from cells. Data are shown as means ± SD, n=3. ^*^P<0.05.

To compare the effects of miRNA targeting HIF-lα on survivin expression, two constructs were prepared and transfected into human A549 cells. After selection for 4 weeks, G418-resistant cells were obtained. Western blotting showed that transfection of the control vector had little effect on HIF-lα expression. However, expression of *HIF-lα* mRNA was markedly downregulated by 67% in cells transfected with HIF-lα-miRNA, and the survivin expression was also downregulated to 75% (Fig. 1B). Western blot analysis showed similar downregulation of HIF-lα and survivin protein expression (Fig. 1A). Furthermore, transfection of survivin expression vectors in HIF-lα knockdown cells rescued survivin expression (Fig. 1). Such an effect was not observed in control cells transfected with empty pCRII-TOPO (data not shown). These results suggest that HIF-lα-miRNA can potently and specifically inhibit endogenous survivin expression in A549 cells.

### Effect of HIF-lα expression on survivin promoter activity in A549 cells

**Figure 2:**
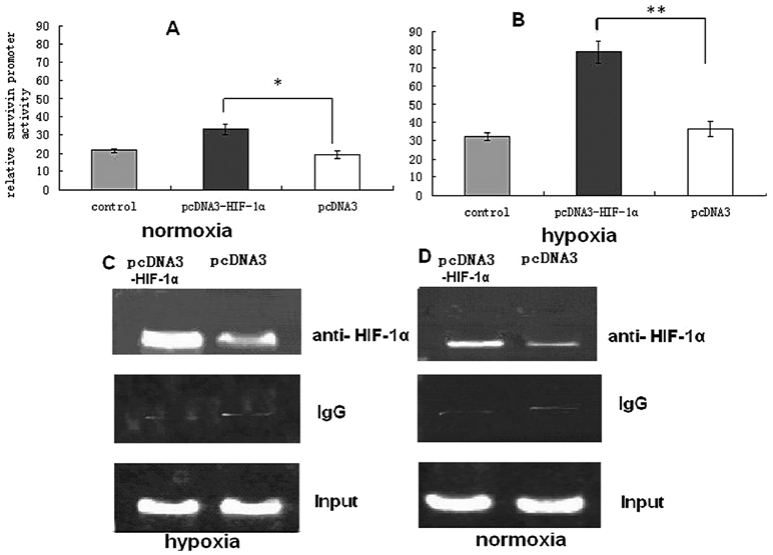
Mechanism of HIF-1α-activated *survivin* gene expression. Cultures were cotransfected with pGL3-SVP-230 and either HIF-1α or control vector in A549 cells under normoxia (A) and hypoxia (B). Relative *survivin* promoter activity was assayed by luciferase. A549 cells were transfected with HIF-1α or empty vector; ChIP assay then confirmed HIF-1α directly binding to *survivin* promoter under hypoxia (C) and normoxia (D). Prior to immunoprecipitation, input was used as an internal control; mouse IgG was used as negative control. Data represent means ± SD (n=3). ^*^P<0.05, ^**^P<0.01, vs. control.

To determine if survivin overexpression is mediated by *HIF-1α* transcriptional activity, we first performed cotransfection experiments under normoxic and hypoxic conditions. Transfection with expression vector pcDNA3-HIF-1α vs. a control led to a 3–4-fold induction of *survivin* promoter activity following cotransfection under hypoxic conditions, which suggests transcriptional regulation of survivin by HIF-1α under hypoxic conditions (Fig. 2B). Although A549 cells transfected with pcDNA3-HIF-1α significantly increased *survivin* promoter activity under normoxic and hypoxic conditions, compared with untreated cells and those transfected with pcDNA3, survivin promoter activity under hypoxic conditions was significantly higher than under normoxic conditions (Fig. 2B). Our previous study detected a putative binding site for HIF-1α, located at ^−^16 to ^−^19 bp in the proximal promoter region of the human *survivin* gene (14). To show HIF-1α binds to the *survivin* promoter in living cells, we performed a ChIP assay in A549 cells under normoxia and hypoxia. In the chromatin fraction pulled down by an anti-HIF-1α antibody, we detected higher expression of *survivin* promoter PCR fragments in pcDNA3-HIF-1α-transfected cells than in pcDNA3-transfected cells under both normoxia and hypoxia (Fig. 2C and D). However, *survivin* promoter PCR fragments were not found in samples pulled down by a control IgG antibody. This further indicated that HIF-1α affects *survivin* transcription by direct binding to an HIF-1α site in the *survivin* core promoter.

### Effect of HIF-lα-miRNA on A549 cell proliferation

**Figure 3:**
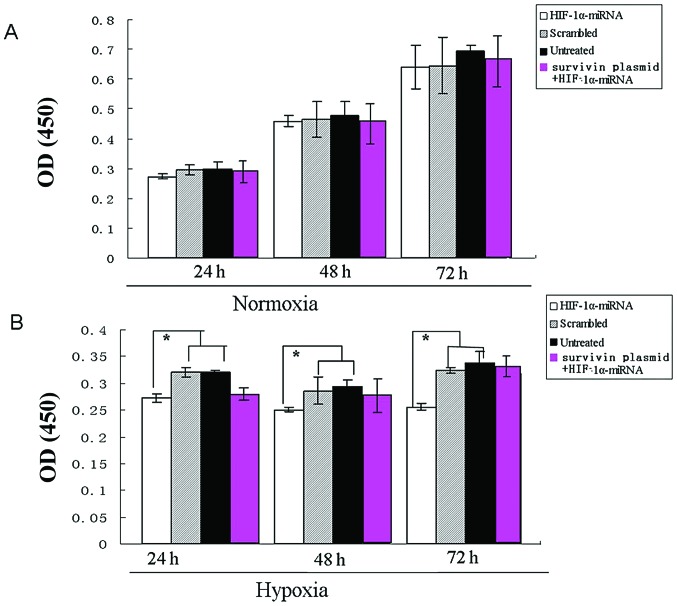
CCK-8 assay. Longitudinal axis shows 450-nm OD microplate value for each well to determine A549 cell viability, for untreated, Scrambled, miRNA plasmid-transfected and survivin plasmid + miRNA groups. Latitudinal axis shows days after cells were treated with or without CoCl_2_ in a 96-well plate under normoxia (A) and hypoxia (B). Data are shown as means ± SD; n=3. ^*^P<0.05.

To verify if specific blockade of HIF-lα inhibits cell proliferation under normoxia and hypoxia, we assayed cell viability in each group of cells transfected with HIF-lα miRNA, Scrambled miRNA (control) or untreated (PBS), for 24, 48 and 72 h. Specific blockade of HIF-lα by miRNA inhibited proliferation at 24 h in A549 cells under hypoxia; following treatment with 150 μmol/l CoCl_2_ for 48 and 72 h, the difference remained (Fig. 3B). Conversely, effects of HIF-1α-miRNA on proliferation did not statistically differ between Scrambled and untreated groups under normoxia (Fig. 3A). These results indicate HIF-lα only promotes lung cancer proliferation under hypoxia, whereas survivin expression vector transfection in HIF-lα knockdown cells partly revived proliferation under hypoxia (Fig. 3).

### Effect of HIF-lα-miRNA on A549 cell apoptosis

**Figure 4:**
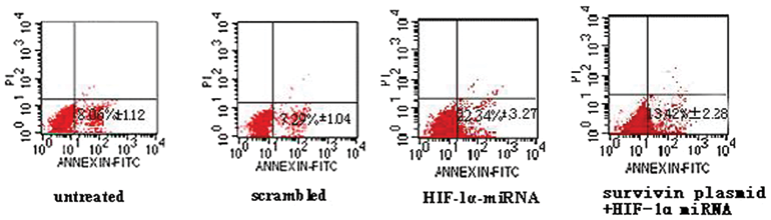
Apoptosis detected by flow cytometry. Apoptosis rate in the HIF-1α-miRNA group was significantly higher than in groups treated with Scrambled, untreated or survivin plasmid + miRNA (P<0.05) under hypoxia; however, the effect of HIF-1α-miRNA on apoptosis under normoxia did not differ significantly between the Scrambled and untreated groups (data not shown).

Apoptosis was analyzed by flow cytometry under both hypoxic and normoxic conditions. The apoptosis ratio of miRNA-transfected cells in hypoxic conditions was 22.34±3.27%, which was significantly higher than in untreated and Scrambled cells (Fig. 4). However, under normoxia, apoptosis rates in the HIF-1α-miRNA group did not statistically differ from those of Scrambled and untreated cells (data not shown). Furthermore, survivin expression vector transfection in HIF-lα knockdown cells rescued the apoptotic phenotype under hypoxic but not under normoxic conditions (Fig. 4).

### Effect of HIF-lα-miRNA on A549 cell invasion

**Figure 5:**
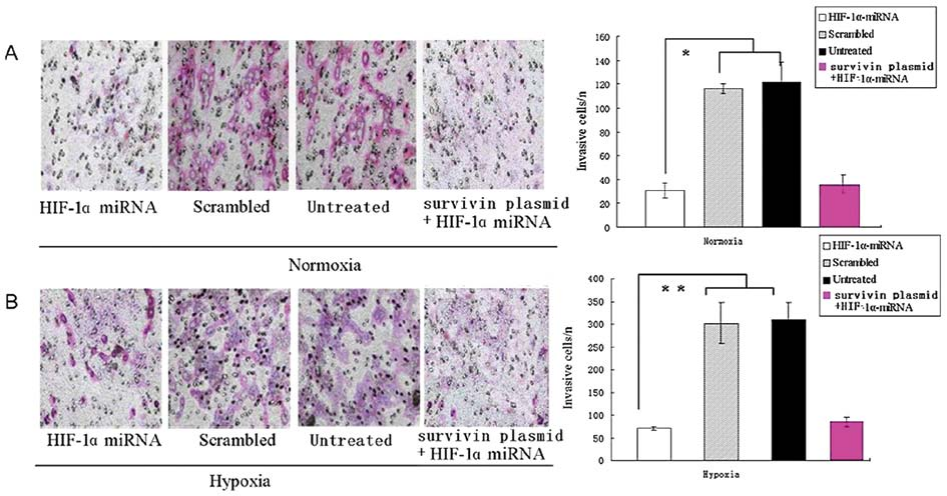
Invasiveness of A549 cells shown by Transwell assay under normoxia and hypoxia. Invasiveness was less in HIF-1α miRNA-transfected cells, as shown by fewer cells invading the lower poly-carbonic membrane surface under normoxia compared with controls (A); invasiveness of treated cells was even less under hypoxia (B). Data are given as means ± SD; n=3. ^*^P<0.05; ^**^P<0.01.

To evaluate the anti-invasive effect of miRNA on A549 cells under normoxic and hypoxic conditions, we used a Transwell assay. Representative micrographs of Transwell filters are shown in Fig. 5. A549 cells transfected with miRNA constructs were significantly less invasive under normoxic and hypoxic conditions, compared with untreated and Scrambled cells (Fig. 5). Meanwhile, the number of invasive cells under hypoxic conditions was significantly higher than under normoxic conditions (Fig. 5). The result suggests that human HIF-lα expression knocked down by miRNA can significantly reduce A549 cell invasion under normoxic and hypoxic conditions, but re-expression of survivin by transfection of expression vectors in the HIF-lα knockdown cells does not promote invasive activity regardless of hypoxic or normoxic conditions (Fig. 5).

### Antitumor effect of HIF-lα-miRNA on an A549 cell xenograft model

**Figure 6:**
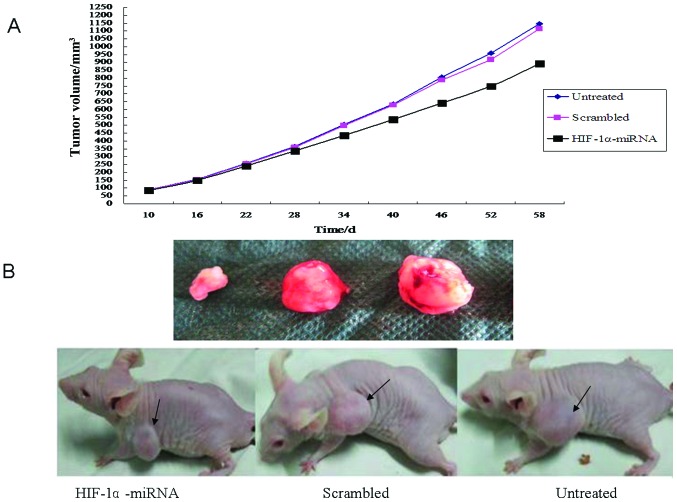
HIF-1α-miRNA resulted in significantly inhibited tumor growth. Mice were inoculated subcutaneously with A549 cells and treated with HIF-1α-miRNA or Scrambled RNA. (A) Growth curves show that tumors of the HIF-1α-miRNA group grew slower than in the untreated and Scrambled groups. (B) Representative images show tumor volume of the HIF-1α-miRNA group was less than in the untreated and Scrambled groups.

To further study the antitumor effect of HIF-lα-miRNA on A549 cells *in vivo*, we used an A549 xenograft model and lipofectamine-mediated gene therapy as indicated in Materials and methods. Tumors were established subcutaneously in the axillary cavities of 24 mice by inoculating cultured cells in the 3 groups. The tumor formation rate in nude mice was 100%. Following inoculation, nodules could be felt subcutaneously in the control and Scrambled groups at 4–5 days, but not until 6–7 days in the HIF-1α-miRNA group. The standard (~50 mm^3^) occurred after 10 days; volume of each tumor was measured by sliding calipers every 3 days. *HIF-lα* gene silencing resulted in statistically significant reduction of tumor volumes compared with the untreated and the Scrambled groups (P<0.01; Fig. 6A). After mice were observed for 58 days, tumor samples were excised and weighed. Tumor weight in the HIF-1α-miRNA group was 1.14±0.08 g, significantly lower than that in the untreated (1.71±0.18 g) and the Scrambled group (1.75±0.26 g) (Fig. 6B, P<0.01), but differences between the untreated and Scrambled groups were not significant (P>0.05).

**Figure 7:**
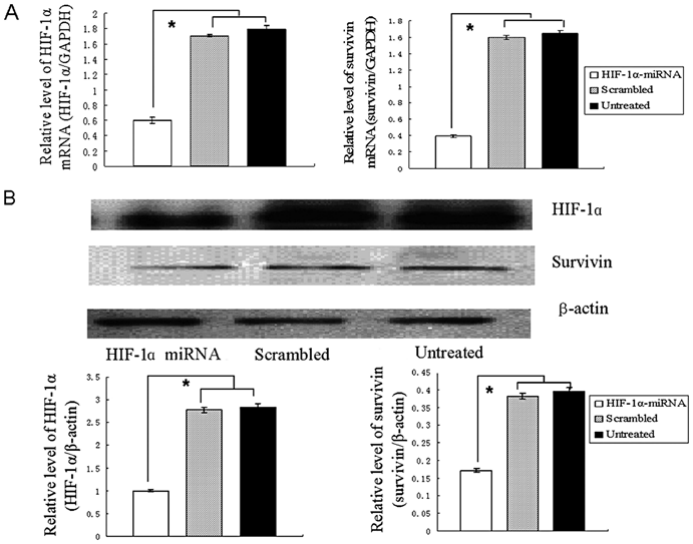
Knocked down HIF-1α expression by miRNA plasmids in transplanted tumor tissue. (A) *HIF-1α* and *survivin* gene expression were measured by RT-PCR using total RNA isolated from transplanted tumor tissue. (B) Western blot analysis of HIF-1α and survivin protein expression using total protein extracted from transplanted tumor tissue. Data are given as means ± SD; n=3. ^*^P<0.05.

**Figure 8:**
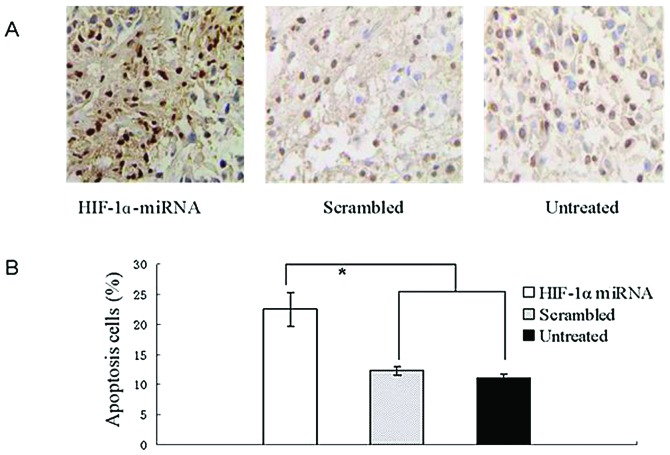
Apoptosis in 3 transplanted tumor tissues, examined by TUNEL assay. (A) Representative images show apoptosis of tumors in HIF-1α-miRNA, Scrambled and untreated groups. (B) Percentage of TUNEL^+^ cell nuclei calculated relative to total number of cell nuclei. Data are given as means ± SD; n=3. ^*^P<0.05.

RT-PCR was used to detect expression of *HIF-1α* mRNA and *survivin* mRNA in tumor tissues (Fig. 7A). Where expression of *HIF-1α* was knocked down by miRNA, survivin expression was significantly lower than in the Scrambled and untreated groups. Western blot results were consistent with the PCR results (Fig. 7B). TUNEL staining showed that apoptosis was prominently increased in the HIF-1α-miRNA group compared with the untreated and Scrambled groups (Fig. 8; P<0.01), Thus, these data show that silencing HIF-1α expression by miRNA significantly inhibits expression of HIF-1α mRNA and protein, and suppresses growth of human pulmonary adenocarcinoma in tumor-bearing nude mice. Decreased survivin expression is responsible for these results. Nude mice in the HIF-1α miRNA group did not differ in body weight gain, feed uptake or locomotive activity from the other groups. No deaths occurred in any groups.

## Discussion

The data presented in this study clearly indicate that HIF-1α mediates survivin expression *in vitro* and *in vivo*. First, assays revealed that inhibition of HIF-1α by miRNA in A549 cells led to decreased survivin expression under normoxia and hypoxia. We next showed that HIF-1α activated the *survivin* promoter by direct interaction with binding sites in the promoter region. In addition, HIF-1α-miRNA induced cell apoptosis and inhibited cell proliferation in A549 cells under hypoxic, but not normoxic, conditions. Cell migration was substantially suppressed by HIF-1α silencing both under normoxia and hypoxia. Transfection of survivin expression vectors in HIF-lα knockdown cells partly rescued the apoptotic phenotype and cell proliferation under hypoxic conditions. By contrast, expression vectors had only slight effect on cell migration. Finally, we confirmed that silencing HIF-1α expression downregulates survivin expression in lung cancer xenografts.

Previous studies have shown that *survivin* promoter activity is significantly increased in tumor cells (16,17). This suggests that survivin expression is transcriptionally regulated. Our recent data suggested that Sp1 strongly affects upregulation of survivin in lung cancer cells at the transcriptional level (18). However, how *survivin* transcription is regulated by other, possibly cis-acting elements is unclear. Notably, a putative HIF-1α binding site lies within the survivin core promoter (19), as confirmed by our previous results, which found site-directed mutagenesis of the HIF-1α binding site reduced *survivin* transcriptional activity by 36.60% (14). The mechanism by which HIF-1α activates survivin expression is unclear. Survivin levels are also strongly upregulated in A549 cells by hypoxia compared with normoxia, as described in our previous study (15,20). This could be explained by the involvement of HIF-1α, (a member of the basic helix-loop-helix-PAS protein family) which is induced predominantly by hypoxia and subsequently translocates into the nucleus where it dimerizes with HIF-1β, consequently regulating a series of gene expression events critical for cellular function under hypoxic conditions (21).

Our study confirmed that HIF-1α and survivin are co-overexpressed in the lung cancer cell line A549. This finding is consistent with studies that show positive rate of HIF-lα is 58.33% and positive rate of survivin is 81.60% in lung cancer tissue, and their expressions correlate with one another (14), indicating that HIF-1α regulates survivin expression. Thus, we tested the impact of HIF-1α on survivin expression in lung cancer cells. As anticipated, our data showed that the silencing of *HIF-1α* by miRNA inhibited survivin expression in A549 cells under hypoxic conditions, which is in accordance with another study showing *HIF-1α* siRNA to block EGF-induced survivin upregulation and to increase apoptosis induced by docetaxel in breast cancer cell lines under normoxia (19). However, our earlier study did not show this effect by transient transfection of *HIF-1α* siRNA in A549 under normoxic conditions. We suspect that different tumor cells and different stimuli may result in HIF-1α showing different effects on *survivin* gene expression under normoxia.

To further investigate the mechanism by which HIF-1α regulates survivin expression, we performed cotransfection experiments under normoxic and hypoxic conditions to test the effect of HIF-1α on *survivin* promoter activity. The *survivin* promoter was markedly activated in A549 cells transfected with pcDNA3-HIF-1α under hypoxic conditions, but was only slightly activated under normoxic conditions, suggesting HIF-1α upregulates survivin expression at the transcription level under hypoxic conditions. Our previous study detected a putative binding site for HIF-1α, located at ^−^16 to ^−^19 bp in the proximal promoter region of the human *survivin* gene (14). In light of this, we used a ChIP assay to determine if HIF-1α can directly bind to the above *survivin* promoter region binding sites, indicating that HIF-1α exerts its effect on the *survivin* promoter by direct interaction, consistent with our previous electrophoretic mobility shift assay (EMSA), which indicated that nuclear extracts of A549 could bind to the r-^32^P-labeled 18-bp probe (nucleotides ^−^26 to ^−^9 of the *survivin* core promoter) which includes binding sites for HIF-1α (22). The mechanism for HIF-1α-mediated transcriptional activation of the *survivin* gene is currently under investigation.

Our laboratory recently demonstrated that HIF-1α cooperated with Notch-1 signaling to increase survivin expression through its direct association with N1ICD, thus accelerating survivin transcription (20). Understanding the molecular mechanism is crucial and urgent for the development of new and improved therapeutic strategies for NSCLC.

Previous findings suggest that survivin is critical to both the initiation of cell proliferation and the inhibition of apoptosis in lung cancer cells. We tested the downstream effects of HIF-1α miRNA on cell growth and apoptosis. Our study showed miRNA-mediated downregulation of HIF-1α expression in A549 cells resulted in significant decline in cell proliferation and increased spontaneous apoptosis under hypoxic conditions. However, these changes did not occur with HIF-1α miRNA under normoxic conditions. This finding is consistent with studies showing HIF-1α to exert anti-apoptotic effects in human umbilical vascular endothelial cells (23), cardiomyocytes (24) and breast cells (19).

Although other studies support our results, Luo *et al* had conflicting observations suggesting that *HIF-1α* siRNA inhibited A549 cell apoptosis by involving the glycolysis pathway (25). Compared with siRNAs used by Luo *et al*, we consider that miRNA used in our experiment silences target genes in vector-infected cells more effectively (26,27). Moreover, in this experiment we designed two other miRNA sequences to confirm that our results were not caused by an off-target effect (data not shown). Also, compared with their study which only utilized transient transfection with *HIF-1α* siRNA plasmids, we adopted both transient transfection and stable transfection methods to ensure silencing effects in the previous and present experiments. Significantly, whereas Luo *et al* did not further investigate the role of HIF-1α in apoptosis *in vivo*, we confirmed that silencing *HIF-1α* gene expression using miRNA can increase apoptosis in nude mice, which has not previously been reported.

To further investigate the effect of survivin on apoptosis induction and cell proliferation inhibition by HIF-1α-miRNA in A549 cells under hypoxic conditions, we transfected survivin expression vectors into HIF-lα-knockdown cells. Re-expression of survivin in the HIF-lα knockdown cells partly revived cell proliferation and rescued the apoptotic phenotype under hypoxic conditions (Fig. 3), indicating that upregulation of survivin is a cause of the protective effects exerted by HIF-1α in A549. Our results also suggested that gene silencing does not affect cell proliferation and apoptosis under normoxia. These may be due to the lower HIF-1α expression under normoxia (which is inadequate to activate survivin), or the dynamic balance between apoptosis and anti-apoptosis signaling pathways regulated by HIF-1α. Our hypotheses are supported by our previous study that expression of HIF-1α and survivin is increased significantly in hypoxia compared with normoxia (15).

Of note, HIF-1α miRNA inhibits A549 cell migration under both normoxia and hypoxia. Furthermore, more cells migrated under hypoxia than under normoxia (data not shown). This result is consistent with that of Shyu *et al*(28), which suggests that HIF-1α overexpression promotes migration of lung cancer cells. However, survivin re-expression in HIF-lα knockdown cells does not promote invasive activity in either hypoxic or normoxic conditions, suggesting that survivin is not related to the effect of HIF-lα on migration. The mechanism by which HIF-1α induces migration warrants further study; establishing a clear link between HIF-1α and survivin in A549 cells could provide new information on the mechanisms by which HIF-1α promotes tumor growth.

We thus evaluated, for the first time, whether *in vitro* effects can be obtained *in vivo* in nude mice bearing A549 cells. We used cells transfected with Scrambled-sequence plasmid and eukaryotic expression plasmid to construct transplanted tumors, indicating that survivin downregulation, tumor inhibition and apoptosis induced by HIF-1α miRNA are in accordance with *in vitro* data. Furthermore, animals in this study presented no mortality from the treatments. Notably, HIF-1α miRNA used in our experiments inhibited tumor growth more effectively than survivin RNA interference, as shown in a previous study (29).

We speculate that HIF-1α also mediates tumor progression by a survivin-independent mechanism; this is supported by evidence that HIF-1α regulates expression of approximately 40 genes, such as angiogenic factors, glucose transporters, glycolytic enzymes, survival and invasion factors, which may be critical for tumor progression (30). The benefits of elucidating the HIF-1α pathway in tumorigenesis may lead to development of novel approaches for the prevention of tumor progression and for lung cancer therapies. Long-term effects of HIF-1α miRNA therapy are currently unknown and require further investigation. Our findings *in vivo*, therefore, both corroborate a possible mechanism for upregulated survivin expression in A549, and provide a basis to target the HIF-1α pathway as a lung cancer therapy.

In conclusion, these results show that silencing *HIF-1α* gene expression using miRNA can increase apoptosis and suppress growth of A549 cells by inhibiting expression of survivin *in vitro* and *in vivo*. This suggests that HIF-1α is an important transcription factor involved in the regulation of survivin expression.
